# Nanoscopic diffusion of water on a topological insulator

**DOI:** 10.1038/s41467-019-14064-7

**Published:** 2020-01-14

**Authors:** Anton Tamtögl, Marco Sacchi, Nadav Avidor, Irene Calvo-Almazán, Peter S. M. Townsend, Martin Bremholm, Philip Hofmann, John Ellis, William Allison

**Affiliations:** 1 Institute of Experimental Physics, Graz University of Technology, 8010 Graz, Austria; 2 Cavendish Laboratory, J. J. Thompson Avenue, Cambridge, CB3 0HE UK; 3 Department of Chemistry, University of Surrey, Guildford, GU2 7XH UK; 4 Material Science Division, Argonne National Laboratory, Argonne, 60439 IL USA; 5 Department of Chemistry and Chemical Biology, Rutgers University, 123 Bevier Road, Piscataway, NJ 08854 USA; 6 Center for Materials Crystallography, Department of Chemistry and iNANO, Aarhus University, 8000 Aarhus, Denmark; 7 Department of Physics and Astronomy, Interdisciplinary Nanoscience Center (iNANO), Aarhus University, 8000 Aarhus C, Denmark

**Keywords:** Reaction kinetics and dynamics, Surface spectroscopy

## Abstract

The microscopic motion of water is a central question, but gaining experimental information about the interfacial dynamics of water in fields such as catalysis, biophysics and nanotribology is challenging due to its ultrafast motion, and the complex interplay of inter-molecular and molecule-surface interactions. Here we present an experimental and computational study of the nanoscale-nanosecond motion of water at the surface of a topological insulator (TI), Bi${}_{2}$Te${}_{3}$. Understanding the chemistry and motion of molecules on TI surfaces, while considered a key to design and manufacturing for future applications, has hitherto been hardly addressed experimentally. By combining helium spin-echo spectroscopy and density functional theory calculations, we are able to obtain a general insight into the diffusion of water on Bi${}_{2}$Te${}_{3}$. Instead of Brownian motion, we find an activated jump diffusion mechanism. Signatures of correlated motion suggest unusual repulsive interactions between the water molecules. From the lineshape broadening we determine the diffusion coefficient, the diffusion energy and the pre-exponential factor.

## Abstract (web summary)

Water molecular motion on surfaces underpins a range of phenomena in nature. The authors resolve the nanoscale-nanosecond motion of water at a topological insulator’s surface by helium spin-echo spectroscopy and computations, reporting hopping among sites and repulsion between water molecules.

## Introduction

Water is ubiquitous in everyday life, yet its nanoscale motion at surfaces is a major challenge to theory, which suffers from the lack of experimental insight^1–9^. The motion of protons, the vibrational dynamics and electronic transitions of water at surfaces usually happens at ultrafast time scales (in the order of femtoseconds)^10^. These processes are accessible with ultrafast optical spectroscopy^11–13^, whereas the interfacial diffusion of molecules typically occurs in the pico- to nanosecond regime and is monitored either in real space using microscopic techniques or in reciprocal space using scattering techniques. However, to make these fast diffusive motions accessible to microscopy studies, the process typically needs to be considerably slowed down. At the same time an intrinsic problem of scanning probe microscopy is that the probes inevitably induce perturbation to the fragile water structure, due to the excitation of tunnelling electrons and the tip–water interaction forces^10,14^.

The studied Bi${}_{2}$Te${}_{3}$ surface is classified as a topological insulator (TI)^15^, a class of materials which exhibit topologically protected metallic surface states (TSS) and an insulating bulk electronic structure^16–18^. The distinct properties of their surface states make TIs promising candidates for possible applications in spintronics and quantum information^17–19^, while surface-dominated transport is currently one of the major objectives on the way to technical applications^20,21^. However, topology can have implications far beyond electronic transport properties and topological materials provide a perfect platform for studying phenomena such as heterogeneous catalysis. Since the TSSs are insensitive to details of the surface such as defects or other kinds of disorder, in contrast to trivial surface states, TIs allow to study processes such as the catalytic activity where the condition of the surface is one of the most important, but also most difficult to describe parameters^22^.

The stability of the electronic structure of TI surfaces upon adsorption has been widely studied due to being crucial for future TI based devices^23–29^, including also the modification of the electronic structure upon adsorption and doping^30–35^. On the one hand, Bi${}_{2}$Se${}_{3}$ reacts with water vapour giving rise to an $n$-doping of the surface^23^. In the case of Bi${}_{2}$Te${}_{3}$ on the other hand, it was shown that water adsorption is less pronounced than the adsorption of oxygen^26,36^, even though the reactivity of water with Bi${}_{2}$Te${}_{3}$ is still under debate^26,27,37^. Except for the influence of adsorbates on the electronic structure, chemistry on TI surfaces has been largely ignored, even though it was shown that TIs hold great potential for sensing applications^38,39^ and exfoliation in liquid environment has been used to obtain nanosheets with unique properties^40–42^. With respect to catalysis, the existence of TSS can modify the catalyst–adsorbate interactions^43^, e.g. acting on the adsorption energy of small molecules irrespective of surface modifications^22^, and effect processes sensitive to the adsorbate binding strength such as hydrogen evolution reactions^44^. It has been suggested that the TSSs can be used as an additional parameter to adjust the catalyst–adsorbate interactions, with the TSS acting as a tunable electron bath^22^.

Given such implications a thorough investigation addressing the interaction of TI surfaces with water is overdue, as is an experimental study about the dynamics and diffusion of adsorbates on TI surfaces in general. Up to now studies about the diffusion and mobility of adsorbates on TI surfaces are solely based on theoretical methods and include the diffusion of metal and alkali metal atoms on Bi${}_{2}$Se${}_{3}$^45,46^ and the diffusion of Pb^47^. Since the kinetics of surface chemical reactions and epitaxial processes used to build advanced TI based structures depends on the mobility of adsorbates, an accurate characterisation of these phenomena and a precise understanding of the diffusion mechanism is crucial.

In this work we provide a detailed insight into the atomic-scale motion of water on the TI surface of Bi${}_{2}$Te${}_{3}$(111). Our experiments provide a unique insight into wetting, friction and physisorption for an important class of materials. Therefore, we use helium-3 spin-echo (HeSE) spectroscopy^48^ which allows to follow the atomic-scale motion of atoms and molecules on surfaces, resolving diffusion processes on timescales from ns to sub-ps. The experimental data is analysed in terms of an analytical model for adsorbate diffusion and compared with density functional theory (DFT) calculations including dispersion corrections. The data provides a measure of the diffusion barrier and coefficient and shows that the motion of water molecules on Bi${}_{2}$Te${}_{3}$(111) occurs by activated hopping on a hexagonal lattice. Our results illustrate that the structure and dynamics of water is determined by an intricate interplay of intermolecular interactions and molecule-surface interactions with signatures of correlated motion due to repulsive interactions between the individual water molecules as further detailed in kinetic Monte Carlo (MC) simulations. Finally, since HeSE is capable of delivering detailed information on the energy landscape during diffusion^49,50^, it allows us to elucidate the physics of energy dissipation at solid–liquid interfaces i.e. the mechanisms in which vibrational (e.g. phonons) and electronic energy (e.g. electron–hole pairs) is transferred between the adsorbates and the surface which eventually governs tribology. We conclude from the observed motion that energy dissipation in nanoscale motion of water on TI surfaces lies in the medium to low-friction regime.

## Results

### Water uptake and dynamics measurements

The tendency of water to aggregate upon adsorption on metallic surfaces is well documented in the literature^4–7,51^. After dosing water on Bi${}_{2}$Te${}_{3}$ at 105 K (to different coverages), diffraction scans do not show any sharp peaks, indicating the absence of long range order and the adsorption of water as amorphous multilayers. Between 105 and 130 K (where desorption starts to become significant, see Supplementary Figs. 2 and 3), we could not detect a diffusion signature. Hence, our diffusion measurements were performed in a temperature range of 135–160 K where dynamics could be clearly resolved, while maintaining a small equilibrium water pressure (around $1{0}^{-9}$ mbar) against desorption and ensuring small water densities which allows interpretation of the dynamics observed.

**Fig. 1:**
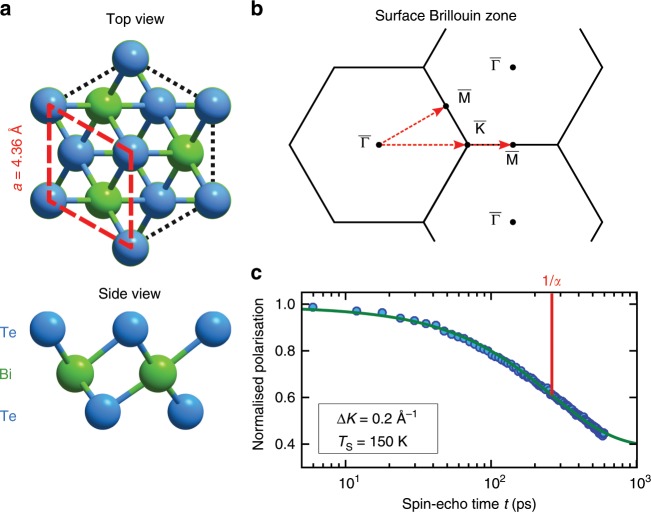
Bi${}_{2}$Te${}_{3}$ structure and dynamics measurements from the lineshape broadening. **a** Top and side view of the (111) surface of Bi${}_{2}$Te${}_{3}$. The surface layer is terminated by a Te layer and the red rhombus highlights the hexagonal surface unit cell. **b** Surface Brillouin zone with the corresponding scanning directions. **c** A typical intermediate scattering function (ISF) showing the normalised polarisation versus spin-echo time $t$. The measured data (filled circles) is fitted with a single exponential decay ((1), solid green line) characterised by the dephasing rate $\alpha$. The logarithmic time axis shows that a single exponential provides a good description of the experimental data.

The dynamics of H${}_{2}$O adsorbed on Bi${}_{2}$Te${}_{3}$ were studied experimentally by measuring the intermediate scattering function (ISF), using a HeSE spectrometer^48^. The ISF, $I(\Delta {\bf{K}},t)$, describes the correlation at the surface after time $t$, for the scattering condition of the helium beam, $\Delta {\bf{K}}$—the parallel momentum transfer. Loss of correlation (dephasing) at $({t}_{1},\Delta {{\bf{K}}}_{1})$, as manifested in the helium beam intensity, is a measure for the dynamics at the surface during time ${t}_{1}$, with the characteristic periodic length scale $2\pi /\Delta {{\bf{K}}}_{1}$. When the adsorbate dynamics are well described as hopping between adsorption sites that form a Bravais lattice, the ISF is known analytically and for a fixed $\Delta {\bf{K}}$ consists of an exponential decay in $t$ with a rate that depends on $\Delta {\bf{K}}$^52^. Allowing for a static offset due to surface defects, $I(\Delta {\bf{K}},t)$ can then be written as:^48,53^

1 $$I(\Delta {\bf{K}},t)={I}_{0}(\Delta {\bf{K}},0)\cdot {\text{e}}^{-\alpha (\Delta {\bf{K}})\cdot t}+C(\Delta {\bf{K}})$$

where $\alpha$ is the dephasing rate and ${I}_{0}$ the amplitude at $t=0$ (see Fig. 1(c) for a typical ISF). The dynamics can then be extracted from the form of $I(\Delta {\bf{K}},t)$ and the functional dependence of $\alpha (\Delta {\bf{K}})$ on $\Delta {\bf{K}}$.

Most dynamics measurements were performed at $150\,{\mathrm{K}}$ at a specular attenuation of ${I}_{0}/3$ and ${I}_{0}/5$ which corresponds to a coverage of 0.05 and 0.08 monolayer (ML), respectively (see Supplementary Methods). The motion of H${}_{2}$O on Bi${}_{2}$Te${}_{3}$(111) was then extracted from HeSE measurements based on a single exponential fit of the experimentally measured ISF according to (1).

At short times, the data shows the expected deviation from a single exponential and we use an iterative routine to optimise the range for inclusion (see Supplementary Methods and Supplementary Fig. 4). Note that the intermediate scattering function may contain information about dynamical processes at different timescales. In particular, the short-range limit typically includes signatures from substrate phonons or may also contain information about intra-cell diffusion^48^. However, in the present work we concentrate on the longer times which are related to the long-range part in diffusion—i.e. the translational part of the diffusion mechanism. The data points within the time-window related to the long-range (translational) diffusion are best fitted with a single-exponential decay and there is no evidence for a double-exponential decay^53^ within this window.

### Diffusion mechanism of H${}_{2}$O

The atomic-scale diffusion of molecules on surfaces is typically described by molecules moving or hopping along the surface while the substrate provides the thermal energy for the motion^48,55–57^. For an activated diffusion process, motion of the adsorbates is governed by the interaction of the molecule with a corrugated potential energy surface (PES). Information about the PES and the hopping motion of the molecule can be obtained from the temperature dependence and the functional form of $\alpha (\Delta {\bf{K}})$^48^.

First, the activation energy for the diffusion of H${}_{2}$O on Bi${}_{2}$Te${}_{3}$(111) can be obtained via temperature dependent measurements at a fixed momentum transfer $\Delta K=| \Delta {\bf{K}}|$. On a not-too-weakly corrugated surface, adsorbate diffusion proceeds by thermally activated hopping whose rate is given by an Arrhenius relation^48,56,58,59^. We will shortly see that $\alpha$ at a fixed $\Delta K$ is proportional to a hopping rate (Fig. 3). Therefore, as long as the diffusion mechanism does not change significantly with temperature, $\alpha ({T}_{S})$ at a fixed $\Delta K$ is also expected to follow an Arrhenius relation:

2 $$\alpha ={\alpha }_{0}\ \exp \left(-\frac{{E}_{a}}{{k}_{B}\ {T}_{S}}\right)$$

where ${\alpha }_{0}$ is the pre-exponential factor describing the jump attempt frequency, ${E}_{a}$ is the activation energy for diffusion, ${k}_{B}$ the Boltzmann constant and ${T}_{S}$ the temperature of the sample surface.

**Fig. 2:**
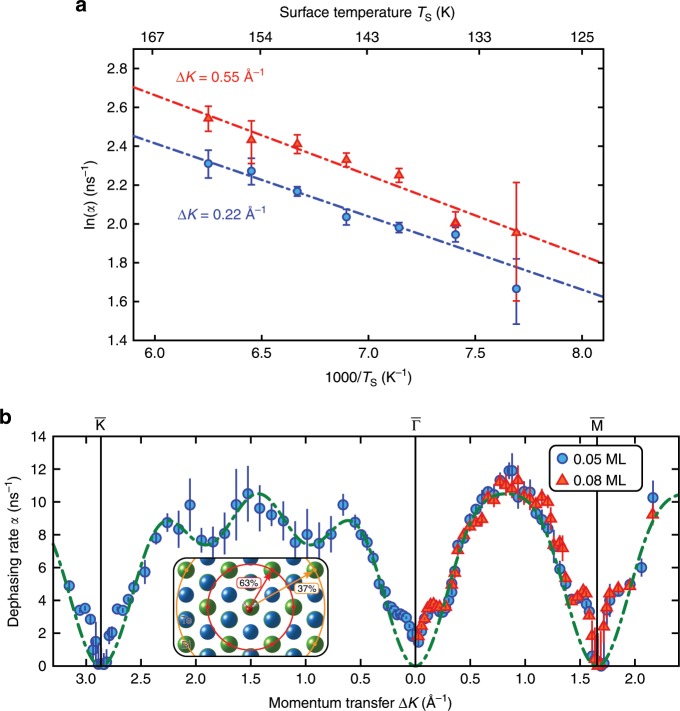
Dephasing rates $\alpha (\Delta K)$ as determined from measurements of $I(\Delta {\bf{K}},t)$. **a** Arrhenius plot showing the temperature dependence at two different momentum transfers along the $\overline{\Gamma {\rm{M}}}$-azimuth for the diffusion of water on Bi${}_{2}$Te${}_{3}$(111). The measurements were taken at an H${}_{2}$O coverage of 0.05 ML. **b** Momentum transfer dependence for the diffusion of H${}_{2}$O on Bi${}_{2}$Te${}_{3}$(111) at a fixed temperature. The measurements were taken at a constant surface temperature of ${T}_{S}=150\,{\rm{K}}$ and a water coverage of 0.05 ML (blue circles) and 0.08 ML (red triangles), respectively. The dash-dotted line corresponds to the analytic model for jump-diffusion with jumps on a hexagonal lattice to nearest and next-nearest sites as illustrated in the small inset. The red and yellow circles illustrate the jump distance to the next and next nearest sites, respectively. The extracted barriers ${E}_{a}$ and pre-exponential factors ${\alpha }_{0}$ for two different momentum transfers $\Delta K$ as well as the pre-exponential hopping rate ${\Gamma }_{0}$ for the diffusion process at 150 K are given in Table 1. The error bars correspond to the confidence bounds ($1\sigma$) upon determination of $\alpha (\Delta K)$ from the measurements of $I(\Delta {\bf{K}},t)$—see text.

Figure 2(a) shows an Arrhenius plot for two different momentum transfers ($\Delta K=0.22$ Å^−1^ and $\Delta K=0.55$ Å^−1^ along $\overline{\Gamma {\rm{M}}}$) over a temperature range from 130 to 160 K. The plot of $\mathrm{ln}(\alpha )$ clearly shows a linear dependence upon $1/{T}_{S}$ as expected for activated motion. To ensure a constant H${}_{2}$O coverage of 0.05 ML at all temperatures, the over-pressure at each temperature was adjusted to maintain an attenuation of the specularly reflected signal by a factor of 3. The uncertainties are the corresponding confidence bounds ($1\sigma$) of the single-exponential fit and the activation energy ${E}_{a}$ is then obtained from the slope of a weighted fit to the Arrhenius plot (Fig. 2(a), see Supplementary Methods for more details), whereupon the intercept gives ${\alpha }_{0}$. Based on both data sets shown in Fig. 2(a) we obtain an activation energy ${E}_{a}$ of:

$${E}_{a}=(34\pm 4)\,{\rm{meV}}.$$

While the temperature range of the measurements is limited by the timescale accessible to the instrument, the range is greater than achieved for water diffusion by some other techniques^2^. There is no evidence of a curvature, which would indicate a temperature dependence of the pre-exponential factor (see Supplementary Note and Supplementary Fig. 6). Only a pathological coincidence where the diffusion mechanism changes in the experimental temperature window would lead to any systematic error in terms of the activation energy or pre-exponential factor.

Having established the diffusion barrier for the motion we now turn to the spatial correlation of the motion. The characteristics of the dephasing rate $\alpha$ versus the momentum transfer $\Delta K$ reflects the underlying energy landscape. The dependence of $\alpha (\Delta K)$ on the momentum $\Delta K$ for the diffusion of H${}_{2}$O on Bi${}_{2}$Te${}_{3}$(111) (${T}_{S}=150\,{\rm{K}}$) is shown in Fig. 2(b) for both high symmetry crystal directions (see Fig. 1(b) for the scanning directions).

Simple hopping motions of an adsorbate can be described by an analytical model, the Chudley–Elliott (CE) model^48,52,55^. It assumes that a particle rests for the mean residence time $\tau$ between motion from one adsorption site to the other. In the case of motion on a Bravais lattice, the dephasing rate $\alpha (\Delta K)$ becomes:

3 $$\alpha (\Delta K)=\frac{2}{\tau }\sum _{n}{p}_{n}{\sin }^{2}\left(\frac{\Delta {\bf{K}}\cdot {{\bf{l}}}_{n}}{2}\right)$$

where ${{\bf{l}}}_{n}$ are the corresponding jump vectors and ${p}_{n}$ is the probability that a jump to the corresponding site occurs.

Figure 2(b) shows that the experimental data can be very well described using a CE model (green dash-dotted line). The best fit using (3) corresponds to jumps on a simple hexagonal Bravais lattice ($a=4.36$ Å) with nearest and next-nearest neighbour jumps, respectively (${p}_{1}=63 \%$, ${p}_{2}=37 \%$). Based on the momentum transfer dependence (Fig. 2(b)), the hopping motion of the water molecules occurs between equivalent adsorption sites, on a hexagonal Bravais lattice with the substrate spacing $a$ (see (Fig. 1(a)).

Any hopping motion between inequivalent adsorption sites would lead to additional decaying exponential components in the tail of the ISF^53^. There is no direct evidence for multi-exponential components in our data, and the fitted $\alpha (\Delta K)$ shows clear zeros at the Bragg condition along both high symmetry directions, where a faster second exponential component would be clearly seen if present^53^. Therefore, the data is entirely consistent with a single relevant adsorption site per primitive cell. In accordance with the results from the vdW corrected DFT calculations (see “DFT Results”), we conclude that jumps occur between the adsorption site above the second layer Bi atoms as shown in the inset of Fig. 2(b).

The corresponding mean residence time of the water molecules in the adsorption sites is $\tau \approx 95\,{\mathrm{ps}}$ based on the CE model. Together with the temperature dependent data (${\alpha }_{0}$ from (2)), the hopping rate from the CE model at 150 K can be related to a pre-exponential factor, now also in terms of a hopping rate ${\Gamma }_{0}=(1.7\pm 0.6)\cdot 1{0}^{11}\,\text{s}^{-1}$. Note that compared to the diffusion of water on other substrates this is at least an order of magnitude smaller^2,60^.

In the region of $\Delta K$ close to zero and around the diffraction peak positions (vertical lines in Fig. 2(b)), the experimental data points lie above the analytical CE model. This is likely to be due to adsorbate-adsorbate interactions which will be discussed below (see “Adsorbate Interactions”). Furthermore, the diffusion coefficient $D$ for two-dimensional motion can be calculated from the hopping rate as determined from the CE model using:

4 $$D=\frac{1}{4}{\left\langle l\right\rangle }^{2}\Gamma$$

where $\Gamma$ is the hopping rate and $\langle l\rangle$ the mean jump length^48,55^. Using the hopping rate at 150 K (Fig. 2(b), $\Gamma =1.05\cdot 1{0}^{10}\,\text{s}^{-1}$) together with the mean jump length of 5.6 Å we obtain a diffusion coefficient of $D=8.2\cdot 1{0}^{-10}\,\text{m}^{2}\ \text{s}^{-1}$.

Compared to the surface diffusion of other small molecules this is rather slow, especially when compared to the diffusion of NH${}_{3}$ on graphite, where an ultra-fast diffusion with $D=3.9\cdot 1{0}^{-8}\,\text{m}^{2}\ \text{s}^{-1}$ was observed at a temperature of 94 K^61^. On the other hand, theoretical studies predict a generally quite small diffusivity on TI surfaces^45^. The reported (ab-initio) diffusion barriers for metal atoms on TI surfaces are typically much larger, in the range of 110–320 meV depending on the adsorbate^45–47^, in line with the predicted small diffusivity. The alkali metal Rb is the only studied adsorbate which shows a diffusivity (as extracted from a kinetic Monte Carlo approach—although at higher temperature^45^) comparable to the one of water found in our study.

### DFT results

We have studied the adsorption of H${}_{2}$O on Bi${}_{2}$Te${}_{3}$ for a number of different adsorption geometries and initial water configurations using van der Waals (vdW) corrected DFT calculations (see “DFT Methods” and Supplementary Fig. 5 for the setup of the supercell). The initial water configurations include both OH bonds pointing down or up, a single OH bond pointing down and a horizontal configuration.

**Fig. 3:**
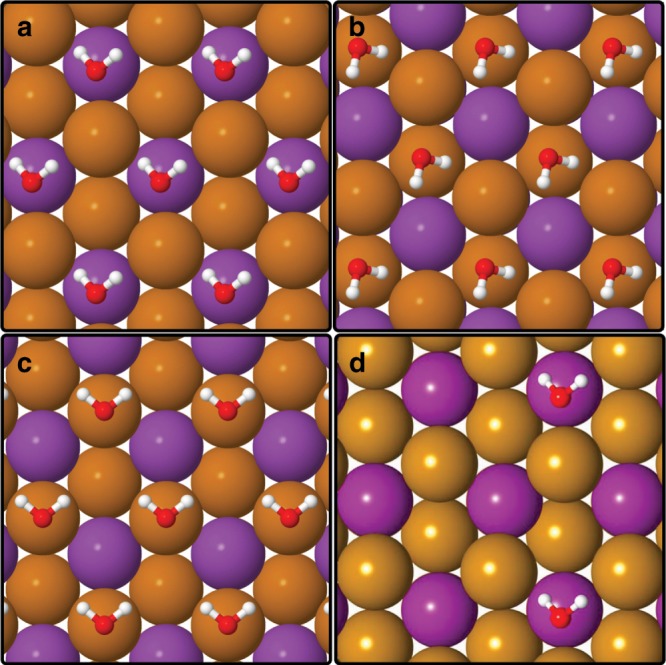
Van der Waals corrected DFT calculations of the adsorption site for H${}_{2}$O on Bi${}_{2}$Te${}_{3}$(111). The Bi and Te atoms are illustrated as purple and brown spheres, respectively. The energetically most favourable adsorption site is on top of the second layer Bi atom (**a**), followed by adsorption on top of the third layer Te atom (**b**) with a difference of 21 meV. Adsorption on top of the first layer Te atom (**c**) is the least favourable adsorption geometry, being 100 meV higher in energy than the absolute minimum. The optimised configuration of the water molecules for a $(2\times 2)$ supercell in **d** is essentially the same as for the higher coverage in **a**.

The adsorption energy of a single water molecule is 271 meV with the H${}_{2}$O molecule at a distance of about 4 Å from the surface. The optimised structures for the minimum energy configuration on the three considered adsorption sites are shown in Fig. 3. The most favourable adsorption site is on top of the second layer Bi atom (Fig. 3a), followed by adsorption above the third layer Te atom (Fig. 3b) with an energy difference of only 21 meV. The first layer Te atom is the least favourable for adsorbing H${}_{2}$O, being 100 meV higher in energy than the absolute minimum. Yashina et al.^26^ reported the third layer Te atom as the most favourable adsorption site, however, they did not consider adsorption above the second layer Bi atom in their study, where we find the largest adsorption energy. In addition, we obtain the same adsorption site and optimised configuration for a $(2\times 2)$ supercell as shown in Fig. 3(d), i.e. when considering a smaller coverage of water molecules on the surface.

Table 2 summarises different orientations of the molecule for adsorption on top of the second layer Bi atom and on the third layer Te atom (for the complete set of DFT calculations including all considered adsorption geometries please refer to the Supplementary Tables 1 and 2). We conclude from Fig. 2 that the optimal orientation of the water molecule on both adsorption sites is at an intermediate angle (skew), i.e. with the OH bond being neither perpendicular nor horizontal to the surface.

**Table 1:** Diffusion parameters as determined from the experimental data in Fig. 2.

$\Delta K$ (Å^-1^)	${E}_{a}$ (meV)	${\alpha }_{0}$ (ns^−1^)
0.22	$32\pm 4$	107 ± 45
0.55	$36\pm 4$	171 ± 72

Extracted diffusion barriers ${E}_{a}$ and pre-exponential factors ${\alpha }_{0}$ for two different momentum transfers $\Delta K$. The pre-exponential hopping rate ${\Gamma }_{0}$ for the diffusion process at 150 K is then ${\Gamma }_{0}=(1.7\pm 0.6)\cdot 1{0}^{11}\,{{\mathrm{s}}}^{-1}$

**Table 2:** Adsorption energies and orientations for water at different adsorption sites on Bi${}_{2}$Te${}_{3}$.

Position	Orientation	${E}_{a}$ (eV)	$\Delta {E}_{a}$ (meV)
1-Te	ho	−0.171	100
1-Te	ld	−0.158	113
2-Bi	skew	−0.271	0
2-Bi	ho	−0.225	46
3-Te	skew	−0.250	21
3-Te	ho	−0.241	29

The adsorption energy ${E}_{a}$ and the energy difference $\Delta {E}_{a}$ relative to the most favourable adsorption site for H${}_{2}$O on Bi${}_{2}$Te${}_{3}$. The table shows adsorption on top of the first layer Te atom (1-Te), the second layer Bi atom (2-Bi) and the third layer Te atom (3-Te). The optimised orientations of the H${}_{2}$O molecule on the according positions are horizontal (ho), with a single OH bond pointing down (ld) or at an intermediate angle (skew)—i.e. with the OH bond being neither perpendicular nor horizontal to the surface

The second most stable adsorption site is where we would approximately localise the diffusion barrier. Based on the results of these static vdW corrected DFT calculations (the temperature of the system is ignored) using the energy differences between the adsorption sites (Fig. 2) would result in a diffusion barrier of about 20 meV, which is in excellent agreement with the value found in the experiment.

As shown in Fig. 3(d), calculations using a $(2\times 2)$ supercell give rise to the same adsorption site as for the $(1\times 1)$ supercell. As can also be seen by a comparison with further results in the Supplementary Table 2, the relative energies and therefore the diffusion barrier do not significantly depend on the water coverage used for the calculations. Hence the experimentally determined barrier for H${}_{2}$O diffusion as well as the one found via vdW corrected DFT are significantly smaller compared to the diffusion barriers of metal atoms on TI surfaces as found by DFT calculations^45–47^.

Since the DFT calculations are static (essentially corresponding to an ideal state at a temperature of 0 K), more evolved theoretical studies such as ab-initio molecular dynamics simulations could address phonons and molecular vibration/rotation effects at finite temperatures but they are computationally extremely demanding for the current system. Furthermore, in most weakly-bound supramolecular systems, zero-point energy effects do not affect substantially the magnitude of the barrier height and are usually ignored since the weak frustrated translation and out-of-plane bending modes are extremely difficult to accurately calculate^61,62^. Hence while the DFT calculations provide a good measure for the energetics of adsorption sites and configurations, for further dynamic properties we rely on other computationally less expensive theoretical approaches as explained below.

### Adsorbate interactions

One reason for the steep rise of $\alpha (\Delta K)$ at small $\Delta K$ values, which is not reproduced by the analytic CE model, may be repulsive interactions between the adsorbates. The characteristic shape of the curve for such a case, with a peak at small $\Delta K$ values followed by a de Gennes narrowing dip, has been described theoretically and observed experimentally for surface diffusion^48,56,58^. The location of the dip corresponds to a peak in the static structure factor^63^, verifies the repulsive nature of the force and allows also a coverage estimation of the adsorbates (see Supplementary Methods).

**Fig. 4:**
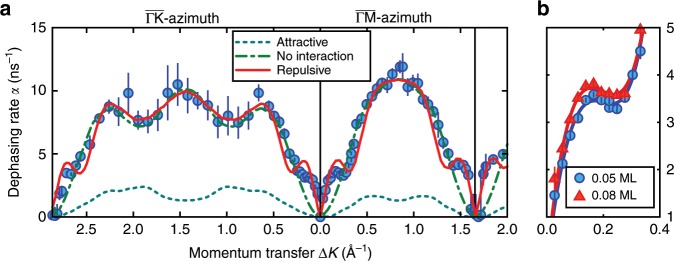
Comparison of the experimental dephasing rates $\alpha (\Delta K)$ with kinetic Monte Carlo simulations and the coverage dependence of $\alpha (\Delta K)$. **a** Momentum transfer dependence $\alpha (\Delta K)$ for the diffusion of H${}_{2}$O on Bi${}_{2}$Te${}_{3}$(111) at 150 K. Experimental results for a coverage of 0.05 ML are shown as discrete points. The red solid line shows $\alpha (\Delta K)$ as extracted from a kinetic Monte Carlo simulation where repulsive dipole–dipole forces between the individual water molecules are included. Attractive interactions (turquoise dashed line) give dephasing rates much lower than the experiment, while diffusion without forces (green dash-dotted line) does not reproduce the structure around the zone centre (see text). **b** A close-up of the experimental dephasing rates in Fig. 2(b) along the $\overline{\Gamma {\rm{M}}}$-direction for two coverages: 0.05 ML, (red line and points), and 0.08 ML (blue line and points). The observed increase in dephasing rate below 0.3 Å^−1^ confirms the presence of repulsive interactions. The error bars correspond to the confidence bounds ($1\sigma$) of $\alpha (\Delta K)$.

The momentum transfer dependence for two different coverages along $\overline{\Gamma {\rm{M}}}$ is shown in Fig. 2(b). $\alpha$ increases slightly when increasing the coverage from 0.05 to 0.08 ML (blue circles vs. red triangles). The difference is quite subtle but seen when zooming into the region at small $\Delta K$ as plotted in Fig. 4(b).

The repulsive nature of the interactions can be verified by a simple kinetic Monte Carlo (MC) simulation, which is illustrated by the red solid line in Fig. 4(a). Therein, we assume that the water molecules move on a hexagonal grid between adjacent sites (based on the results of the analytical model above). Repulsive/attractive inter-adsorbate interactions were included with a pairwise dipole–dipole potential. Using the trajectories of the MC simulation, the dephasing rate $\alpha$ is then determined from the calculated ISFs (see Supplementary Methods for more details). For no interaction between the molecules we obtain the same $\alpha (\Delta K)$ from the MC simulation as the CE model (green dash-dotted line). Attractive interactions between the molecules cannot explain the de Gennes dip (turqouise dashed line). Only the introduction of repulsive forces in the MC simulation can reproduce the experimental data including also the de Gennes dip at small $\Delta K$ and around the diffraction peaks as illustrated by the red solid line in Fig. 4(a). The effect is however much less pronounced compared to other systems e.g. to Na on Cu(001)^58^ and to the diffusion of water on the hydrophobic graphene surface^60^.

The repulsive forces will increase the hopping rate in the long-range limit (for $| \Delta K\pm G|$ approaching 0), i.e. if one adsorbate moves it pushes the other ones in the vicinity due to the repulsive forces, thus increasing the overall hopping rate. The situation of the individual water molecules depends on the distance between the adsorbates and the actual configuration at the surface. Consequently, the dipole moment associated with an adsorbate used in the simulations that describe the experimental data provides a good measure of the interactions. Using a dipole moment of about 2 debye in the MC simulations provides a good description of the experimental data, which corresponds roughly to the dipole moment of an isolated water molecule and is by a factor of three smaller than compared to the Na/Cu(001) system^58^.

### Energy dissipation and atomic-scale friction

Based on the low corrugation of the PES (from experiment and DFT calculations) in combination with the rather small diffusion constant we may already anticipate that the system exhibits an unusual atomic-scale friction. Friction in surface diffusion processes can be caused by a variety of dissipative mechanisms, interactions with phonons and electrons in the substrate^54^ as well as interactions between adsorbates^64^ and coupling of the internal molecular degrees of freedom with the motion of the centre of mass^65^. For Brownian motion, the atomic-scale friction $\eta$ can be directly extracted with Einstein’s relation^61,66^, while it is not possible for hopping motion. In principle the CE model for hopping motion contains Brownian diffusion as a long range diffusion limit (for $\Delta K\to 0$, $\alpha (\Delta K)$ converges to a parabola), but this approach is problematic^61^, in particular for the interacting case. Furthermore, we are not able to resolve any frustrated translational vibration of the water molecule, thus allowing conclusions about the friction force from the broadening of the vibrational mode^56^.

Instead, we base our analysis on the rate of barrier crossing obtained from a Langevin description of the dynamics, where the friction is a direct measure of the coupling between the centre-of-mass motion and the heat-bath of the substrate^67^. Friction has a direct influence on the diffusion as it affects the rate of energy transfer between the adsorbate and the substrate. In the present study we seek to understand the relatively low rate of diffusion noted above. In ideal Brownian diffusion, where there are no barriers, the rate decreases as the friction increases. However, if the diffusion is activated, as in the present case, then there exists also a low-friction regime where the rate decreases as the friction is reduced.

The phenomenology is well understood from a theoretical perspective, where it is known as Kramer’s turnover theory^68^. Motion in the high-friction regime is dominated by single-jumps, while behaviour in the low-friction regime includes both single- and multiple-jumps, though single-jumps always dominate. Comparison with the analysis in Fig. 2(b), and in particular the significant fraction of double jumps, suggest that the observed motion lies in the low-friction regime. Notably, the finding differs from the observation of other molecular adsorbates which generally showed a higher friction^65^.

Understanding the energy dissipation channels during diffusion on TI surfaces is interesting due to their insulating interior, so that the only contribution to electronic friction arises from the metallic surface state. There is no simple way to disentangle the electronic from the phononic contribution to the friction^54^ but it is noteworthy that Bi${}_{2}$Te${}_{3}$ has a low Debye temperature with phonons having correspondingly low frequencies^69,70^. The energy mismatch between the acoustic phonons and the internal modes of a water molecule, together with a mismatch between the mass of the water molecule and the heavy substrate atoms, suggests that phononic friction will occur predominantly through multi-phonon processes (see also Supplementary Discussion and Supplementary Table 3). The likely absence of single-phonon coupling suggests that electronic friction may therefore be a significant contribution. Electron–hole excitation in a metallic band is the predominant mechanism for electronic friction^54^. In the present instance any contribution is restricted to the density of surface states arising from the topological character of the substrate.

Taken together, these observations are suggestive of a system where both the phonon and electron contributions are limited. The picture is consistent with the experimental results in Fig. 2(b), where a high proportion of multiple-jumps is required to explain the data. The newly available data may provide the necessary experimental benchmarks in order to study these effects from a theoretical point of view.

Future work will be required to resolve the importance of the above mentioned effects towards energy dissipation and to explain the measurements fully; in particular considering the full-dimensional potential of the diffusing molecule together with possible internal degrees of freedom of the H${}_{2}$O molecules^65,71^. Other routes for first-principle theoretical calculations regarding the frictional forces are possible within the harmonic approximation and have been applied in the past for calculations, especially for atomic self-diffusion. However, its validity for weakly-bonded supramolecular systems is questionable, especially in the context of a well-known multidimensional and temperature-dependent contribution to the friction force from surface phonons, excitons and other non-adiabatic effects^72^. Furthermore, more evolved theoretical studies such as ab-intio molecular dynamics simulations are on the verge of what is currently possible in terms of computing time considering the observed relatively slow diffusion process on a rather complex surface.

## Discussion

In summary, to our knowledge, this work reveals for the first time the mechanism of water diffusion on a TI surface based on experiments. Our analysis and understanding of the correlated motion of H${}_{2}$O on Bi${}_{2}$Te${}_{3}$(111) provides a more general insight into the mobility of small molecules at TI surfaces. The diffusion of water molecules on Bi${}_{2}$Te${}_{3}$(111) follows an activated hopping motion on a corrugated potential energy surface, with a diffusion barrier of 34 meV, in good agreement with the results of vdW-corrected DFT calculations. Jumps of the water molecules occur on a hexagonal lattice corresponding to the substrate lattice spacing, with a significant fraction of longer jumps (37%).

The mechanism is remarkable as it shows signatures of repulsive interaction between the individual water molecules. The experimentally determined diffusion coefficient is 2–3 orders of magnitude larger than the theoretically calculated atomic mobilities of most metal atoms on TI surfaces, yet slower than the diffusion of small molecules on flat metal surfaces. In addition to the experimental insight into wetting, friction and physisorption of water on an important class of materials, the hereby studied system provides also a special platform for an atomic level investigation in what ways kinetic and chemical energy is transferred between adsorbates and the substrate—not least due to the insulating interior and the existence of topologically protected metallic surface states on TIs.

The observed diffusive properties differ strongly from those at low-index metal surfaces for molecular diffusion on solid surfaces. The experimental data obtained during this study provides the necessary benchmarks for further theoretical progress since experimental and theoretical findings suggest that energy dissipation between the water adsorbates and surface atoms governs the diffusion mechanism.

## Methods

### Experimental details

All measurements were performed on the Cambridge helium-3 spin-echo apparatus which generates a nearly monochromatic beam of ${}^{3}$He that is scattered off the sample surface in a fixed 44.4° source–target–detector geometry. The detailed setup of the apparatus has been described in greater detail elsewhere^48,50^. The crystal structure of Bi${}_{2}$Te${}_{3}$ is rhombohedral, consisting of quintuple layers bound to each other through weak van der Waals forces which gives easy access to the (111) surface by cleavage^15^. The (111) cleavage plane (Fig. 1a) is terminated by Te atoms and exhibits a hexagonal structure ($a=4.386$ Å^21^). The Bi${}_{2}$Te${}_{3}$ single crystals used in the study were attached onto a sample holder using electrically and thermally conductive epoxy. The sample holder was then inserted into the chamber using a load-lock system^73^ and cleaved in situ. The sample holder can be heated using a radiative heating filament on the backside of the crystal or cooled down to 105 K via thermal connection to a liquid nitrogen cooling reservoir. The sample temperature was measured using a chromel-alumel thermocouple.

Water was dosed onto the sample with a microcapillary array beam doser which was brought close to the surface. Previous to the dynamics measurement H${}_{2}$O is dosed up to a certain attenuation (corresponding to a certain H${}_{2}$O coverage, see Supplementary Fig. 1) of the specularly reflected helium signal. Therefore the partial pressure of water in the scattering chamber is adjusted using an automatic leak valve and the reflected helium signal is monitored until equilibrium is obtained.

The dynamics of H${}_{2}$O adsorbed on Bi${}_{2}$Te${}_{3}$(111) were extracted from helium spin-echo measurements, via the intermediate scattering function (ISF), $I(\Delta {\bf{K}},t)$ with a single exponential decay according to (1). While a signal exponential decay provides the best fit to the data throughout the experiments presented here, note that for different types of motion occurring on different timescales $I(\Delta {\bf{K}},t)$ will deviate from the single exponential form in (1) and is better fitted using multiple exponential decays^48^.

### Computational details

For the DFT calculations presented in this work we employed CASTEP^74^, a plane wave, periodic boundary condition code. The plane wave basis set is truncated at an electron energy cut-off of 1000 eV and we employ fully-relativistic pseudopotentials. The Brillouin zone was sampled with a $(5\times 5\times 1)$ Monkhorst–Pack $k$-point mesh. The Perdew–Burke–Ernzerhof (PBE) exchange-correlation functional^75^ was applied in combination with the Tkatchenko–Scheffler (TS) dispersion correction method^76^. The electronic structure was minimised to the ground state energy by fully including spin-orbit coupling (SOC) and non-collinear spin treatment. Due to the topologically non-trivial nature of Bi${}_{2}$Te${}_{3}$ the inclusion of SOC leads to the formation of the TSS, i.e. the Dirac cones in the surface electronic structure^69^. The Bi${}_{2}$Te${}_{3}$ substrate was modelled with a single quintuple layer in a $(1\times 1)$ supercell, and an additional 25 Å vacuum layer for separating the periodically repeated supercells in the $z$-direction. The positions of the atoms in the substrate and adsorbate were left fully unconstrained, except for the set of calculations with the frozen substrate. For the structural optimisations, the force tolerance was set to $0.05\,{\mathrm{eV}}{\mathring{\rm{A}} }^{-1}$.

## Supplementary information

Supplementary Information

## Data Availability

The data that support the findings of this study are available from the corresponding author upon reasonable request.

## Code availability

The code for the kinetic Monte Carlo simulations is available from 10.5281/zenodo.3531646 under the GNU/GPL-3.0 license.
