# Supplementary material for: Nanoscopic diffusion of water on a topological insulator
Source: Nat Commun. 2020 Jan 14;11:278. doi: 10.1038/s41467-019-14064-7 (PMC6959239; doi:10.1038/s41467-019-14064-7)
Supplement: Supplementary file 1 — Supplementary Information [file 41467_2019_14064_MOESM1_ESM.pdf]

Supplementary Information
Nanoscopic diffusion of water on a topological insulator

Tamtögl *et al.*

1. SUPPLEMENTARY METHODS

1.1. Water Adsorption, Coverage and Thermal Desorption

We have carried out adsorption and desorption measurements of H_2O on $Bi_2Te_3(111)$. The processes of adsorption and desorption were observed by following in real time the specular beam intensity of He atoms scattered from the crystal surface during the deposition of water. In order to calibrate the coverage and to investigate the adsorption of H_2O on $Bi_2Te_3(111)$, the helium specular signal I was measured while dosing H_2O at different surface temperatures. The upper panel in figure Supplementary Figure 1 shows the relative He specular peak height I/I_0 as a function of H_2O exposure. Surface exposure is defined as the impinging flux of H_2O on the surface integrated over the time of exposure.
At a temperature of 105 K the intensity of the specular peak falls off sharply, corresponding to the commencement of adsorption and diffuse scattering from the adsorbates. The specular intensity decays almost to zero which is typical for the absence of any ordered structure. This is confirmed by subsequent diffraction experiments which do not show any diffraction signal. We interpret it as being due to the formation of amorphous solid water (ASW) at the surface.
The fact that the presence of H_2O on the surface substantially attenuates the specular beam can be used as a measure of the H_2O coverage. The normalised specular intensity I/I_0 can be related to the H_2O coverage Θ via:

$$I/I_0 = (1-\Theta)^{n\cdot\Sigma/\cos\vartheta_i} \tag{1}$$
$$\approx 1-\Theta\cdot n\cdot\Sigma/\cos\vartheta_i \quad \text{for} \quad \Theta \ll 1 \tag{2}$$

where n is the adsorbate density at monolayer (ML) coverage, Σ is the helium scattering cross section and the term $\cos(\vartheta_i)$ accounts for the increase of the apparent scattering cross section since scattering happens at an incident angle $\vartheta_i = 22.2°$.
The adsorbate density n is given by the monolayer coverage, where one monolayer corresponds to one water molecule per surface unit cell. In case of low coverage, a linear dependence of the intensity on the coverage can be assumed (equation 2) and the scattering cross section Σ can be determined from the initial slope of the adsorption curve.

The different slopes in Supplementary Figure 1a suggest that either the helium scattering cross section changes with temperature or the sticking coefficient decreases with temperature. Since it is unlikely that Σ changes with surface temperature we assume that the sticking coefficient becomes smaller with increasing surface temperature.
The bottom panel in figure Supplementary Figure 1 shows I/I_0 versus coverage where the surface coverage Θ has been determined from the exposure using a Langmuir adsorption model where the sticking coefficient is given as $S(\Theta) = (1-\Theta)S_0$, with S_0 the initial sticking coefficient for the uncovered surface. Here we assume that S_0 decreases with temperature in a way that the initial slope and hence the scattering cross section remains constant. The extracted temperature dependence of S_0 is plotted in Supplementary Figure 2.
From the initial slope we obtain a scattering cross section of ($\Sigma = 260$ Å^2) for isolated water molecules on $Bi_2Te_3(111)$. While this is a high scattering cross it is not unusual for He scattering of isolated adsorbates[1] and also very similar to the gas phase cross sections of water and water-deuterium[2].
We note also that the uptake curve for 150 K in Supplementary Figure 1a shows a sign for repulsive interactions between the adsorbates: The uptake curve falls slightly below the black linear dash-dotted line. This is due to the fact that the scattering cross sections of repelling adsorbate particles overlap less, increasing the total cross section seen by the He beam compared to that for non-interacting adsorbates and thus the uptake curve falls below the linear line.

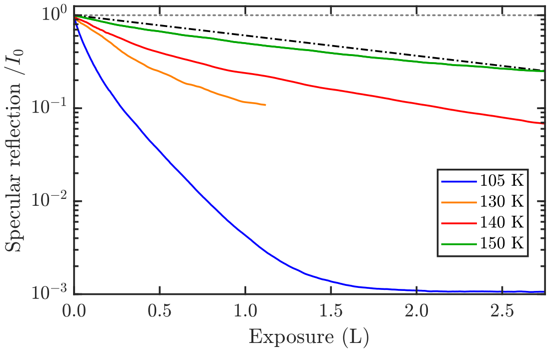

(a) Normalised He reflection versus H_2O exposure.

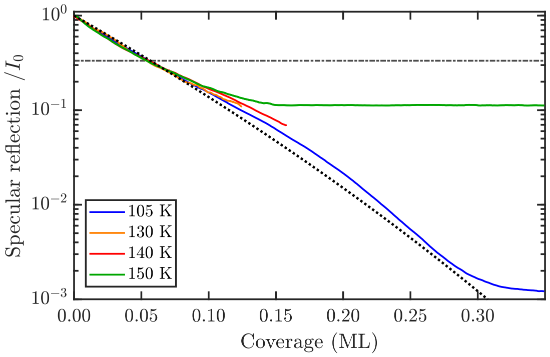

(b) Normalised He reflection versus H_2O coverage, assuming a constant scattering cross section.

Supplementary Figure 1. Top panel: Normalised specular reflection versus exposure for the adsorption of water on $Bi_2Te_3(111)$ at 4 different surface temperatures and an incident beam energy of 8 meV.
Bottom panel: I/I_0 versus coverage plotted on a logarithmic scale. The coverage has been determined from the exposure using the initial sticking coefficients as described in the text. The dashed line corresponds to equation 1 with a scattering cross section $\Sigma = 260$ Å^2.

From an exponential fit to the temperature dependence of the initial sticking coefficient S_0 (Supplementary Figure 2) we see that S_0 becomes very small at room temperature, in the region of low 10^{-5}. This finding is in very good agreement with an early adsorption study by Haneman[3] suggesting that the $Bi_2Te_3(111)$ surface is relatively inert with respect to water adsorption at room temperature.

On the other hand, the reactivity of water with Bi_2Te_3 is still under debate. While Yashina *et al.* conclude that water does not react with Bi_2Te_3 using X-ray photoelectron spectroscopy (XPS) and angle-resolved photoemission spectroscopy (ARPES)[4], Zhang *et al.* reported that water reacts with Bi_2Te_3 eventually giving rise to a surface modification where the surface is terminated by hydrated Bi bilayers and Bi bilayer islands[5].

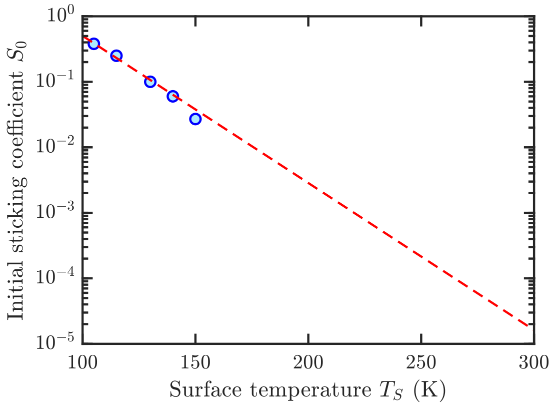

Supplementary Figure 2. Temperature dependence of the initial sticking coefficient S_0 for the adsorption of water on $Bi_2Te_3(111)$. An extrapolation based on an exponential fit (red dashed line) suggests that the sticking coefficient at room temperature is very low, of the order of 10^{-5}).

In general, our adsorption and diffraction measurements support the findings of Yashina *et al.*[4]. During our experiments the adsorption of water on Bi_2Te_3 is a completely reversible process, i.e. upon heating above the desorption temperature, water will completely desorb leaving the clean crystal behind as confirmed by the same specularly scattered intensity. Furthermore, from diffraction measurements we can exclude any structural changes to the surface after adsorption and desorption of water. On the other hand the lowest adsorption temperature in our study was 105 K, while Zhang *et al.* observe the formation of H_2Te only at temperatures of 100 K, including also much larger H_2O doses[5] compared to our study.

We have also conducted thermal desorption spectroscopy (TDS) measurements while monitoring the m/z = 18 peak on a mass spectrometer and simultaneously measuring the specularly reflected He signal. Supplementary Figure 3 shows a typical thermal desorption measurement after water had been deposited on $Bi_2Te_3(111)$ at a surface temperature of 105 K. Clearly visible is one dominant peak with a maximum at 167 K which coincides with a rapid recovery of the specular signal (right axis in Supplementary Figure 3). The Redhead equation can be applied, in order to estimate the desorption energy E_{des}. Redhead's equation reads as:[6]

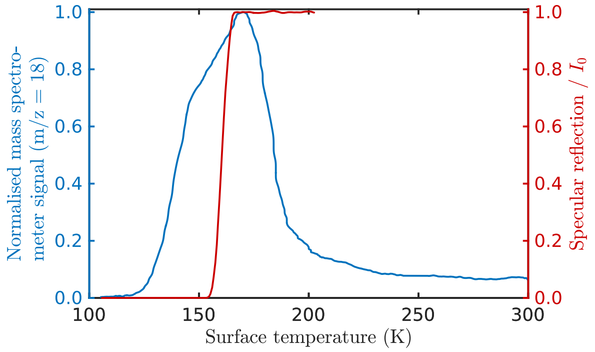

Supplementary Figure 3. Thermal desorption spectroscopy (left axis) together with the specular He reflection (right axis) for water deposited on $Bi_2Te_3(111)$ at a temperature of 105 K.

$$E_{des} = k_B \cdot T_m \left[\ln \left(\frac{\nu \cdot T_m}{\beta} \right) - 3.46 \right], \qquad (3)$$

with T_m the temperature of the desorption maximum, k_B the Boltzmann constant, β the heating rate and the preexponential or frequency factor ν. With $T_m = 167$ K, $\beta = 0.1$ K^{-1} and $\nu = \Gamma_0$ (as determined in the main part of the manuscript) we obtain a desorption energy of $E_{des} = (0.43 \pm 0.03)$ eV.

Note that the desorption energy is larger than the adsorption energy of a single water molecule obtained from the vdW corrected DFT calculations. However, the TDS was taken after depositing water at 105 K, giving rise to a surface which is fully covered by amorphous solid water. Hence it is likely that the adsorption energy in ASW is larger compared to an isolated water molecule on the surface. Indeed the experimentally determined value of E_{des} is close to the sublimation enthalpy of ice with 0.49 eV[7].

Most dynamics measurements have been performed at an attenuation of $I_0/3$ which is illustrated by the dash-dotted line in Supplementary Figure 1b and corresponds to a coverage of about 0.05 ML. For a constant dosing pressure at 150 K the He signal levels off after a certain exposure. The system is thus, in an adsorption-desorption equilibrium. While with increasing overpressure the coverage increases, with increasing surface temperature the dynamic equilibrium is reached faster. Within the available temperature range - where we could observe diffusion and where we are able to obtain a constant coverage by applying an overpressure - it was found that measurements at 150 K provided the best trade-off in order to clearly see dynamics and maintain constant experimental conditions.

We can also use the position of the de-Gennes narrowing (dip) to estimate the coverage. In accordance to cal-

culating a scaled (1×1) diffraction pattern in reciprocal space, the position of the de-Gennes dip is given by $\Delta K_{dip} = 4\pi/\sqrt{3}r$ where r is the preferred spacing among the adsorbates in this quasi-hexagonal structure. The coverage Θ is then given via:

$$\Theta = \frac{3\,a^2\,\Delta K_{dip}^2}{16\pi^2} \tag{4}$$

where a is the surface lattice constant of $Bi_2Te_3(111)$ and ΔK_{dip} the momentum transfer at which the de-Gennes narrowing occurs. Based on the position of the dips at the two highest coverages in Figure 4a we obtain $\Theta = 0.03$ ML for $I_0/3$ and $\Theta = 0.04$ ML for $I_0/5$. The values are somewhat smaller than those obtained form the uptake curves but since (4) can only serve as an approximation it shows that the estimation of the coverage is consistent.

1.2. Analysis of the ISFs

As described in the main part of the manuscript the experimentally measured intermediate scattering function (ISF) $I(\Delta\mathbf{K}, t)$ was fitted with a single-exponential decay:

$$I(\Delta\mathbf{K}, t) = I_0(\Delta\mathbf{K}, 0) \cdot \mathrm{e}^{-\alpha(\Delta\mathbf{K})\cdot t} + C(\Delta\mathbf{K}) \tag{5}$$

where α is the dephasing rate and I_0 the amplitude at $t = 0$ for a typical ISF.
In the present work we concentrate on the timescales which are related to the long-range part in diffusion - i.e. the translational part of the diffusion mechanism. Since the short-range limit typically includes signatures from substrate phonons or may also contain information about intra-cell diffusion[8], the data points at very small times are excluded from the fit, using an iterative routine to find the optimum exclusion limit.
The approach is based on changing the exclusion limit while looking at the R^2 of the fit (see Supplementary Figure 4), which is an established method for spin-echo measurements[9]. At large cut-off times, the quality of the fit is limited by the lack of data and the R^2 value starts small. As the number of included data points increases, α stabilises at around 11 ns^{-1} and R^2 increases, indicating that the model represents the data. For times smaller than 10 ps, R^2 decreases since the quantity of the data not described by the exponential model increases, which is consistent with the onset of a faster decay process at very short timescales.

1.3. Fitting and Uncertainties

The measured ISFs were fitted in MATLAB using the "trust-region-reflective" algorithm, in order to determine the dephasing rate α. The corresponding uncertainties of the dephasing rates are obtained according to the confidence bounds for the fitted coefficients (1-σ).

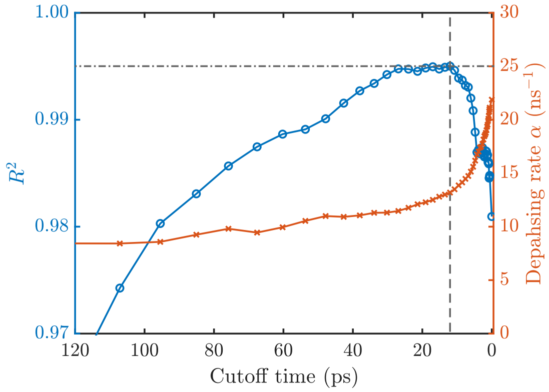

Supplementary Figure 4. Sensitivity of the time cut-off, when optimising an exponential model $I_0 \cdot \mathrm{e}^{-\alpha\cdot t} + C$ to the experimental data. Blue circles show the R^2 coefficient of the fit while red crosses correspond to the value of the obtained dephasing rate α as a function of the cut-off time. At large cut-off times the fit to the exponential is weak and at very small times the influence of a faster decay distorts the result.

The data in the Arrhenius plot is again fitted using the "trust-region-reflective" algorithm, including the uncertainties of the obtained dephasing rates as weights. The uncertainties of the activation energy and the preexponential factor are then those obtained from the confidence bounds of the fitted coefficients, i.e. the slope and the intercept of Fig. 2(a) in the main part of the manuscript. The uncertainty for the preexponetial factor in terms of the hopping rate (Γ_0), is obtained via propagation of uncertainties.

1.4. Kinetic Monte Carlo Simulations

Monte Carlo (MC) simulations employing a modified form of the Metropolis algorithm were used to give an insight into the mechanism of adsorbate interactions during diffusion[10,11]. The code for the kinetic MC simulations is available from `https://doi.org/10.5281/zenodo.3531646` under the GNU/GPL-3.0 license.
In the MC simulation water molecules can move on a hexagonal lattice with jumps up to second nearest neighbour sites. A periodic (60×40) grid was used in the simulations, where H_2O molecules were initially put down on the grid in turn at random. The potential energy for an adsorbate at each site of the grid was calculated for the initial configuration, taking into account inter-adsorbate interactions. Inter-adsorbate interactions are modelled in the MC simulations by adding/subracting the following term to the potential energy surface:

$$\frac{\pm A}{r^3} = \frac{p^2}{4\pi\epsilon_0 r^3} \tag{6}$$

where p is the effective value of the dipole moment and r is the distance separating the two dipoles and $\pm$ accounts for repulsive/attractive interactions.

An MC event consists of choosing an adsorbate at random which may then hop to one of its neighbouring sites, with different probabilities for jumps to first and second nearest neighbours. Provided that the water molecule is not blocked from entering the new site by another molecule the probabilities are weighted by the difference in the potential of the molecule at the two sites. If several new sites with lower potential energy exist, one of them is chosen at random and the molecule is moved into the new site. The trajectories of the molecules versus time $\mathbf{R}_n(t)$ obtained from the MC simulation are then used to calculate the (ISF):

$$I(\Delta\mathbf{K},t) = \frac{1}{N}\sum_{m,n}^{N}\left\langle e^{i\Delta\mathbf{K}(\mathbf{R}_m(t)-\mathbf{R}_n(0))}\right\rangle \qquad (7)$$

with N, the total number of particles. The ISFs obtained from the simulation are then analysed in the same way as the experimental data: The ISF is fitted with a single exponential decay which allows to determine the dephasing rate $\alpha(\Delta K)$ from the simulation in analogy to the curve determined from the experiments.

Supplementary Table 1. The adsorption energy E_a and the energy difference ΔE_a relative to the most favourable adsorption site for H_2O on Bi_2Te_3. The table shows adsorption on top of the first layer Te atom (1-Te), second layer Bi atom (2-Bi) and the third layer Te atom (3-Te). The In column defines the starting point for the orientation with both OH bonds pointing down (td) or up (up), a single OH bond pointing down (ld) and a horizontal configuration (ho). The out column gives the optimised orientation of the H_2O molecule on the according positions, with either horizontal (ho) or at an intermediate angle (skew) - i.e with the OH bond being neither perpendicular nor horizontal to the surface.

Position	E_a (eV)	ΔE_a (meV)	In	Out
1-Te	−0.018	253	td	td
1-Te	−0.008	263	up	up
1-Te	−0.171	100	ho	ho
1-Te	−0.158	113	ld	ld
2-Bi	−0.271	0	td	skew
2-Bi	−0.225	46	up	ho
2-Bi	−0.225	46	ho	ho
2-Bi	−0.262	9	ld	skew
3-Te	−0.241	29	td	ho
3-Te	−0.231	40	up	ho
3-Te	−0.220	51	ho	ho
3-Te	−0.250	21	ld	skew

2. SUPPLEMENTARY TABLES

In this section we present the data for all adsorption geometries / configurations of water on $Bi_2Te_3(111)$ which have been calculated using van der Waals corrected DFT. Supplementary Figure 5 shows the setup of the (2×2) supercell for the simulations, illustrating also the molecular orientations which refer to the orientation of the OH bonds in the plane of the C_2 axis.

Supplementary Table 1 shows the adsorption energies

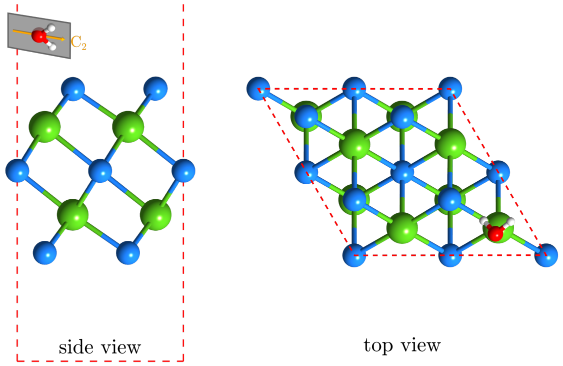

Supplementary Figure 5. Illustration of a top and side view of the (2×2) unit cell (red dashed line) used for the vdW corrected DFT calculations. Note that the full vacuum spacing in the side view is not shown. The labelling of the molecular orientation refers to the orientation of the OH bonds in the plane of the C_2 axis (orange arrow in the side view) with respect to the surface plane.

for three different positions together with the orientation of the water molecule before (In) and after the optimisation (Out). Supplementary Table 2 compares the results for a (1×1) supercell with a (2×2) supercell while in Supplementary Table 3 the adsorption energies for the frozen substrate are given.

The DFT computations where performed with inclusion of fully relativistic pseudopotentials and inclusion of spin-orbit coupling (SOC) corrections and hence the DFT calculation is fully able to predict the correct band structure of the topological insulator, i.e. the topological surface states (TSS) are present in the electronic dispersion. The obtained electronic band structure is in agreement with previously reported experimental and theoretical studies, therefore we believe that our modelling work correctly describes the topological nature of the substrate in its interaction with water.

Supplementary Table 2. Comparison of the energy difference ΔE_a relative to the most favourable adsorption site for a (2×2) and a (1×1) supercell. The nomenclature of the position and orientation is the same as in Supplementary Table 1.

Position	(1×1) cell ΔE_a (meV)	(1×1) cell Out	(2×2) cell ΔE_a (meV)	(2×2) cell Out
1-Te	100	ho	104	ho
2-Bi	0	skew	0	skew
3-Te	21	skew	20	ld

Supplementary Table 3. The adsorption energy E_a and the energy difference ΔE_a relative to the most favourable adsorption site for H_2O on Bi_2Te_3 for a frozen substrate. The nomenclature of the position and orientation is the same as in Supplementary Table 1.

Position	E_a (eV)	ΔE_a (meV)	In	Out
1-Te	−0.031	231	td	td
1-Te	−0.009	252	up	up
1-Te	−0.170	92	ho	ho
1-Te	−0.169	92	ld	ld
2-Bi	−0.224	37	td	ho
2-Bi	−0.235	27	up	ho
2-Bi	−0.219	42	ho	ho
2-Bi	−0.261	0	ld	skew
3-Te	−0.237	24	td	ho
3-Te	−0.231	30	up	ho
3-Te	−0.223	38	ho	ho
3-Te	−0.249	12	ld	skew

3. SUPPLEMENTARY NOTE

Modified Arrhenius: Since accurate experimental estimates of the pre-exponential factor are often missing, several theoretical studies make use of a pre-factor $k_B T/h$ without further refinements[12,13]. If experimental data is available an extension of the simple Arrhenius equation can be written in terms of a pre-exponential factor α_0 which is proportional to T^n with a constant n according to:

$$\alpha = B \cdot T^n \exp\left(-\frac{E_a}{k_B T}\right) , \quad (8)$$

where B is is a temperature-independent constant. Equation 8 results in a curved temperature dependence of $\alpha(T)$ for $n \neq 0$.

The result of fitting various pre-exponential functional

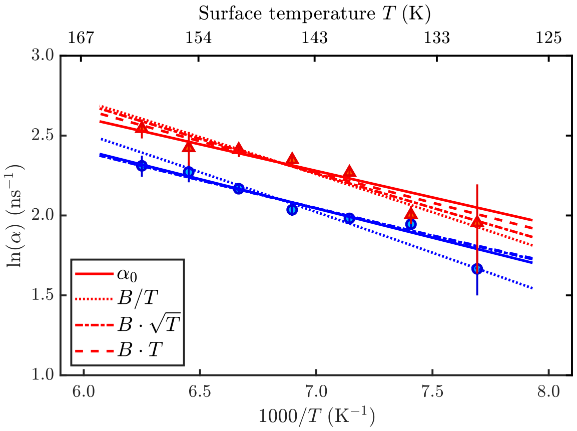

Supplementary Figure 6. Arrhenius plot for the diffusion of water on Bi_2Te_3(111), fitted with different modified Arrhenius equations according to (8).

forms that give a curved Arrhenius plot is shown in Supplementary Figure 6. We note that the goodness of fit for Equation 8 with $-1/2 \leq n \leq 1$ is comparable and the determined diffusion barrier E_a is largely unaffected by the choice of n. Thus the effect of using a modified version of the Arrhenius with a temperature dependent pre-exponential factor $B \cdot T^n$ causes only a slightly increased uncertainty of E_a for $-1/2 \leq n \leq 1$ and we can safely assume a linear dependence within the studied temperature window.

4. SUPPLEMENTARY DISCUSSION

Phononic Friction: Energy dissipation at surfaces and interfaces is mediated by excitation of elementary processes, including phonons and electronic excitation[9,14,15]. As mentioned in the main part of the manuscript contributions from energy dissipation via phonons as well as electronic effects can be expected for the diffusion on Bi_2Te_3. The low lying acoustic surface phonon modes[16,17] will already be occupied at the thermal energies of the measurements while at the same time the metallic surface states of Bi_2Te_3 should account for electronic effects. The phononic contribution can in principle be estimated using the framework of an elastic continuum model and the harmonic approximation[11,14] and this approach would give for example a phononic contribution in the the case of water on Bi_2Te_3 that is much larger than the one estimated for benzene on graphite[18]. We performed also vdW corrected DFT calculations of H_2O on Bi_2Te_3 with a frozen substrate, which gave rise to a smaller barrier (12 meV) as reported in Supplementary Table 3. While this result does not necessarily prove that phonons are involved in the diffusion process, it certainly suggests that both phononic and electronic effects contribute to the atomic-scale friction.

Understanding the energy dissipation channels during diffusion on TI surfaces is particularly interesting due to their insulating interior and the possible role of topological surface states to act as a tunable "electron bath"[19]. E.g. changing the electronic part of the friction may have a significant impact as observed on superconducting surfaces: Friction encountered in atomic force microscopy on superconducting materials typically shows a strong decrease below the critical temperature[20] which is ascribed to the suppression of electronic friction in the superconducting state[15,20].

The concept of phononic friction on metallic surfaces on the other hand, is eventually a matter of relaxation times[15,21]: energy dissipated by friction always goes ultimately to phonons, but on metal surfaces it is mostly transmitted to phonons via electron-phonon (e-ph) interaction, at least for non-polarisable species which do not have appreciable direct interaction with surface atom cores. Thus for closed shell adsorbates with negligible polarisability, the phononic friction is mainly determined by how fast the electron gas transfers the surplus energy to phonons, i.e., the strength of the e-ph

interaction. For adsorbed polarisable objects like heavy atoms or molecules the problem is more complicated because there is the competition of the direct transfer to phonons without the mediation of the free-electron gas. Thus the obtained data provides the necessary experimental benchmarks in order to study these effects from a theoretical point of view.

SUPPLEMENTARY REFERENCES

[1] Farías, D. & Rieder, K.-H. Atomic beam diffraction from solid surfaces. *Rep. Prog. Phys.* **61**, 1575 (1998).

[2] Mammoli, D. *et al.* Collisional cross-section of water molecules in vapour studied by means of 1H relaxation in NMR. *Sci. Rep.* **6**, 38492 (2016).

[3] Haneman, D. Adsorption and bonding properties of cleavage surfaces of bismuth telluride. *Phys. Rev.* **119**, 567 (1960).

[4] Yashina, L. V. *et al.* Negligible Surface Reactivity of Topological Insulators Bi_2Se_3 and Bi_2Te_3 towards Oxygen and Water. *ACS Nano* **7**, 5181–5191 (2013).

[5] Zhang, K.-W. *et al.* Real-space characterization of reactivity towards water at the Bi_2Te_3(111) surface. *Phys. Rev. B* **93**, 235445 (2016).

[6] Redhead, P. Thermal desorption of gases. *Vacuum* **12**, 203–211 (1962).

[7] Eisenberg, D. & Kauzmann, W. *The structure and properties of water* (Oxford University Press, 2005).

[8] Jardine, A., Hedgeland, H., Alexandrowicz, G., Allison, W. & Ellis, J. Helium-3 spin-echo: principles and application to dynamics at surfaces. *Prog. Surf. Sci.* **84**, 323 (2009).

[9] Rittmeyer, S. P. *et al.* Energy dissipation during diffusion at metal surfaces: Disentangling the role of phonons versus electron-hole pairs. *Phys. Rev. Lett.* **117**, 196001 (2016).

[10] Metropolis, N., Rosenbluth, A. W., Rosenbluth, M. N., Teller, A. H. & Teller, E. Equation of State Calculations by Fast Computing Machines. *J. Chem. Phys* **21**, 1087–1092 (1953).

[11] Ala-Nissila, T., Ferrando, R. & Ying, S. C. Collective and single particle diffusion on surfaces. *Adv. Phys.* **51**, 949–1078 (2002).

[12] Honkala, K. *et al.* Ammonia Synthesis from First-Principles Calculations. *Science* **307**, 555–558 (2005).

[13] Ellis, J. & Toennies, J. P. Observation of jump diffusion of isolated sodium atoms on a Cu(001) surface by helium atom scattering. *Phys. Rev. Lett.* **70**, 2118–2121 (1993).

[14] Persson, B. Sliding friction. *Surf. Sci. Rep.* **33**, 83–119 (1999).

[15] Park, J. Y. & Salmeron, M. Fundamental Aspects of Energy Dissipation in Friction. *Chem. Rev.* **114**, 677–711 (2014).

[16] Tamtögl, A. *et al.* Nanoscale surface dynamics of Bi_2Te_3(111): observation of a prominent surface acoustic wave and the role of van der Waals interactions. *Nanoscale* **10**, 14627–14636 (2018).

[17] Chis, V. *et al.* Vibrations in binary and ternary topological insulators: First-principles calculations and Raman spectroscopy measurements. *Phys. Rev. B* **86**, 174304 (2012).

[18] Hedgeland, H. *et al.* Measurement of single-molecule frictional dissipation in a prototypical nanoscale system. *Nature Phys.* **5**, 561–564 (2009).

[19] Schoop, L. M., Pielnhofer, F. & Lotsch, B. V. Chemical Principles of Topological Semimetals. *Chem. Mater.* **30**, 3155–3176 (2018).

[20] Kisiel, M. *et al.* Suppression of electronic friction on Nb films in the superconducting state. *Nat. Mater.* **10**, 119 (2011).

[21] Askerka, M., Maurer, R. J., Batista, V. S. & Tully, J. C. Role of tensorial electronic friction in energy transfer at metal surfaces. *Phys. Rev. Lett.* **116**, 217601 (2016).
